# 5-Bromo-2,4,6-trimethyl-3-methyl­sulfinyl-1-benzofuran

**DOI:** 10.1107/S1600536808032467

**Published:** 2008-10-15

**Authors:** Hong Dae Choi, Pil Ja Seo, Byeng Wha Son, Uk Lee

**Affiliations:** a Department of Chemistry, Dongeui University, San 24 Kaya-dong, Busanjin-gu, Busan 614-714, Republic of Korea; b Department of Chemistry, Pukyong National University, 599-1 Daeyeon 3-dong, Nam-gu, Busan 608-737, Republic of Korea

## Abstract

In the title compound, C_12_H_13_BrO_2_S, there are two symmetry-independent mol­ecules, *A* and *B*, in the asymmetric unit. The crystal studied was an inversion twin with a 0.70 (2):0.30 (2) domain ratio. The methyl­sulfinyl group in mol­ecule *B* is disordered over two positions with site-occupancy factors fixed at 0.6 and 0.4. The crystal structure is stabilized by C—H⋯O hydrogen bonds and inter­molecular C—H⋯π inter­actions. In addition, the crystal structure exhibits C—Br⋯π inter­actions, with C—Br⋯*Cg*1 = 3.646 (8) Å where *Cg*1 is the centroid of the furan ring.

## Related literature

For the crystal structures of similar 3-methyl­sulfinyl-1-benzofuran compounds, see: Choi *et al.* (2007*a* ▶,*b* ▶).
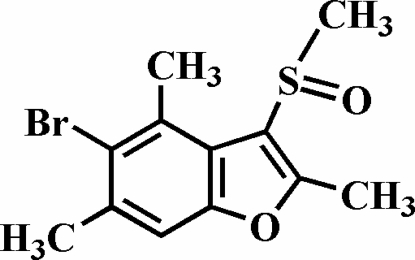


## Experimental

### 

#### Crystal data

C_12_H_13_BrO_2_S
*M* *_r_* = 301.19
Monoclinic, 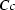

*a* = 28.128 (4) Å
*b* = 11.013 (1) Å
*c* = 8.052 (1) Å
β = 102.290 (2)°
*V* = 2437.1 (5) Å^3^
*Z* = 8
Mo *K*α radiation
μ = 3.53 mm^−1^
*T* = 298 (2) K
0.40 × 0.40 × 0.30 mm

#### Data collection

Bruker SMART CCD diffractometer
Absorption correction: multi-scan (*SADABS*; Sheldrick, 1999 ▶) *T*_min_ = 0.269, *T*_max_ = 0.345
7147 measured reflections
4360 independent reflections
3737 reflections with *I* > 2σ(*I*)
*R*_int_ = 0.020

#### Refinement

*R*[*F* ^2^ > 2σ(*F* ^2^)] = 0.042
*wR*(*F* ^2^) = 0.112
*S* = 1.09
4360 reflections
317 parameters
2 restraints
H-atom parameters constrained
Δρ_max_ = 0.45 e Å^−3^
Δρ_min_ = −0.90 e Å^−3^
Absolute structure: Flack (1983 ▶), 1736 Friedel pairs
Flack parameter: 0.70 (2)

### 

Data collection: *SMART* (Bruker, 2001 ▶); cell refinement: *SAINT* (Bruker, 2001 ▶); data reduction: *SAINT*; program(s) used to solve structure: *SHELXS97* (Sheldrick, 2008 ▶); program(s) used to refine structure: *SHELXL97* (Sheldrick, 2008 ▶); molecular graphics: *ORTEP-3* (Farrugia, 1997 ▶) and *DIAMOND* (Brandenburg, 1998 ▶); software used to prepare material for publication: *SHELXL97*.

## Supplementary Material

Crystal structure: contains datablocks I. DOI: 10.1107/S1600536808032467/sj2545sup1.cif

Structure factors: contains datablocks I. DOI: 10.1107/S1600536808032467/sj2545Isup2.hkl

Additional supplementary materials:  crystallographic information; 3D view; checkCIF report

## Figures and Tables

**Table 1:** Hydrogen-bond geometry (Å, °) *Cg*2 is the centroid of the C2–C7 benzene ring.

*D*—H⋯*A*	*D*—H	H⋯*A*	*D*⋯*A*	*D*—H⋯*A*
C12—H12*A*⋯O2^i^	0.96	2.34	3.298 (9)	179
C12—H12*C*⋯O4*B*^ii^	0.96	2.42	3.168 (12)	134
C24*A*—H24*A*⋯O4*A*^iii^	0.96	2.35	3.199 (13)	148
C9—H9*A*⋯*Cg*2^iii^	0.96	3.30	3.943 (9)	124

Symmetry codes: (i) 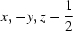; (ii) 
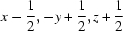
; (iii) 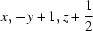.

**Fig. 1.**
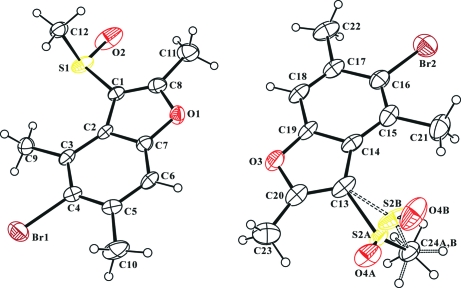
The asymmetric unit of the title compound, showing displacement ellipsoids drawn at the 30% probability level. Bonds to atoms of the major disorder component of the disordered methylsulfinyl group are drawn as double dashed lines.

**Fig. 2.**
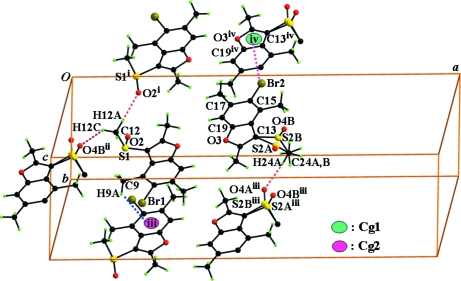
C—H···O, C—H···π and C—Br···π interactions (dotted lines) in the title compound. Cg denotes a ring centroid. [Symmetry code: (i) x, -y, z - 1/2; (ii) x - 1/2, -y + 1/2, z + 1/2; (iii) x, -y + 1, z + 1/2; (iv) x, -y, z - 1/2.]

## supplementary crystallographic information

### Comment

This work is related to our previous communications on the synthesis and structure of 3-methylsulfinyl-1-benzofuran analogues, *viz*. 5-bromo-2-methyl-3-methylsulfinyl-1-benzofuran (Choi *et al.*, 2007*a*) and 2,5-dimethyl-3-methylsulfinyl-1-benzofuran (Choi *et al.*, 2007*b*). Here we report the crystal structure of the title compound, 5-bromo-2,4,6-trimethyl-3-methylsulfinyl-1-benzofuran which crystallizes with two unique molecules, A & B in the asymmetric unit (Fig. 1).

The benzofuran unit is essentially planar, with a mean deviation of 0.012 (6) Å for A and 0.0022 (7) Å for B, respectively, from the least-squares plane defined by the nine constituent atoms. The crystal studied was an inversion twin with a 0.70 (2):0.30 (2) domain ratio.  The O4=S2—C24H_3_ group in molecule B is disordered over two positions with site-occupancy factors fixed at 0.4 (for atoms labelled A) and 0.6 (for atoms labelled  B) in Fig. 1.  The crystal structure is stabilized by three intermolecular C—H···O hydrogen bonds (Fig. 2 and Table 1; symmetry codes as in Fig. 2). The molecular packing (Fig. 2) is further stabilized by a intermolecular C—H···π interactions, with a C9—H9A···*Cg*2^iii^ separation of 3.30 Å (Fig. 2 and Table 1; *Cg*2 is the centroid of the C2—C7 benzene ring, symmetry code as in Fig. 2). In addition, the molecular packing exhibits an intermolecular C—Br···π interaction between the Br atom and the furan ring, with a C16—Br2···*Cg*1^iv^ separation of 3.646 (8) Å (Fig. 2; *Cg*1 is the centroid of C13—C14/C19/O3/C20 furan ring, symmetry code as in Fig. 2).

### Experimental

3-Chloroperoxybenzoic acid, 77% (197 mg, 0.88 mmol) was added in small portions to a stirred solution of 3-methylsulfanyl-2-phenyl-1-benzofuran (228 mg, 0.8 mmol) in dichloromethane (30 ml) at 273 K. After stirring for 3 h at room temperature, the mixture was washed with saturated sodium bicarbonate solution and the organic layer was separated, dried over magnesium sulfate, filtered and concentrated under vacuum. The residue was purified by column chromatography (ethyl acetate) to afford the title compound as a colorless solid [yield 79%, m.p. 400–401 K; *R*_f_ = 0.61 (ethyl acetate)]. Single crystals suitable for X-ray diffraction were prepared by evaporation of a solution of the title compound in acetone at room temperature. Spectroscopic analysis: ^1^H NMR (CDCl_3_, 400 MHz) δ 2.51 (s, 3H), 2.68 (s, 3H), 2.83 (s, 3H), 2.95 (s, 3H), 7.21 (s, 1H); EI—MS 302 [*M*+2], 300 [*M*^+^].

### Refinement

All H atoms were geometrically positioned and refined using a riding model, with C—H = 0.93 Å for aromatic H atoms, 0.96 Å for methyl H atoms, respectively, and with *U*_iso_(H) = 1.2Ueq(C) for aromatic H atoms and 1.5Ueq(C) for methyl H atoms.

The crystal studied was an inversion twin with a 0.70 (2):0.30 (2) domain ratio. The O4=S2—C24H_3_ group in molecule B is disordered over two positions with site-occupancy factors fixed at 0.40 (for atoms labelled A) and 0.60 (for atoms labelled B) in the final refinement. Atomic and anisotropic displacement parameters of C24A and C24B were restrained using the commands EXYZ and EADP.

### Crystal data

**Table:**

C_12_H_13_BrO_2_S	*F*(000) = 1216
*M**_r_* = 301.19	*D*_x_ = 1.642 Mg m^−^^3^
Monoclinic, *C**c*	Melting point = 401–400 K
Hall symbol: C -2yc	Mo *K*α radiation, λ = 0.71073 Å
*a* = 28.128 (4) Å	Cell parameters from 3249 reflections
*b* = 11.013 (1) Å	θ = 2.9–26.1°
*c* = 8.052 (1) Å	µ = 3.53 mm^−^^1^
β = 102.290 (2)°	*T* = 298 K
*V* = 2437.1 (5) Å^3^	Block, colorless
*Z* = 8	0.40 × 0.40 × 0.30 mm

### Data collection

**Table:**

Bruker SMART CCD diffractometer	4360 independent reflections
Radiation source: fine-focus sealed tube	3737 reflections with *I* > 2σ(*I*)
graphite	*R*_int_ = 0.020
Detector resolution: 10.0 pixels mm^-1^	θ_max_ = 27.0°, θ_min_ = 1.5°
φ and ω scans	*h* = −35→28
Absorption correction: multi-scan (SADABS; Sheldrick, 1999)	*k* = −14→12
*T*_min_ = 0.269, *T*_max_ = 0.345	*l* = −9→10
7147 measured reflections	

### Refinement

**Table:**

Refinement on *F*^2^	Secondary atom site location: difference Fourier map
Least-squares matrix: full	Hydrogen site location: inferred from neighbouring sites
*R*[*F*^2^ > 2σ(*F*^2^)] = 0.042	H-atom parameters constrained
*wR*(*F*^2^) = 0.112	*w* = 1/[σ^2^(*F*_o_^2^) + (0.0294*P*)^2^ + 6.0617*P*] where *P* = (*F*_o_^2^ + 2*F*_c_^2^)/3
*S* = 1.09	(Δ/σ)_max_ = 0.001
4360 reflections	Δρ_max_ = 0.45 e Å^−^^3^
317 parameters	Δρ_min_ = −0.90 e Å^−^^3^
2 restraints	Absolute structure: Flack (1983), 1736 Friedel pairs
Primary atom site location: structure-invariant direct methods	Flack parameter: 0.70 (2)

### Special details

**Table:**

Geometry. All e.s.d.'s (except the e.s.d. in the dihedral angle between two l.s. planes) are estimated using the full covariance matrix. The cell e.s.d.'s are taken into account individually in the estimation of e.s.d.'s in distances, angles and torsion angles; correlations between e.s.d.'s in cell parameters are only used when they are defined by crystal symmetry. An approximate (isotropic) treatment of cell e.s.d.'s is used for estimating e.s.d.'s involving l.s. planes.
Refinement. Refinement of *F*^2^ against ALL reflections. The weighted *R*-factor *wR* and goodness of fit *S* are based on *F*^2^, conventional *R*-factors *R* are based on *F*, with *F* set to zero for negative *F*^2^. The threshold expression of *F*^2^ > σ(*F*^2^) is used only for calculating *R*-factors(gt) *etc*. and is not relevant to the choice of reflections for refinement. *R*-factors based on *F*^2^ are statistically about twice as large as those based on *F*, and *R*- factors based on ALL data will be even larger.

### Fractional atomic coordinates and isotropic or equivalent isotropic displacement parameters (Å^2^)

**Table:**

	*x*	*y*	*z*	*U*_iso_*/*U*_eq_	Occ. (<1)
Br1	0.21922 (3)	0.71294 (6)	0.66785 (9)	0.0659 (2)	
Br2	0.50554 (3)	−0.18594 (8)	0.33653 (9)	0.0854 (3)	
S1	0.17522 (7)	0.15294 (19)	0.6928 (2)	0.0577 (5)	
S2A	0.5460 (2)	0.4107 (6)	0.3537 (10)	0.0523 (16)	0.40
O4A	0.5400 (5)	0.4962 (11)	0.2811 (18)	0.067 (4)	0.40
C24A	0.5884 (2)	0.3483 (7)	0.5252 (9)	0.0616 (19)	0.40
H24A	0.5790	0.3678	0.6298	0.092*	0.40
H24B	0.6201	0.3813	0.5265	0.092*	0.40
H24C	0.5893	0.2617	0.5126	0.092*	0.40
S2B	0.5499 (2)	0.3607 (6)	0.3149 (7)	0.081 (2)	0.60
O4B	0.5586 (3)	0.3734 (12)	0.1963 (14)	0.099 (4)	0.60
C24B	0.5884 (2)	0.3483 (7)	0.5252 (9)	0.0616 (19)	0.60
H24D	0.5945	0.4278	0.5740	0.092*	0.60
H24E	0.6187	0.3109	0.5173	0.092*	0.60
H24F	0.5724	0.2996	0.5957	0.092*	0.60
O1	0.30391 (17)	0.2256 (4)	0.5845 (6)	0.0485 (11)	
O2	0.1830 (2)	0.0201 (6)	0.7294 (9)	0.097 (3)	
O3	0.42047 (19)	0.2988 (4)	0.4257 (7)	0.0614 (14)	
C1	0.2298 (2)	0.2149 (5)	0.6484 (9)	0.0366 (15)	
C2	0.2427 (2)	0.3433 (5)	0.6439 (7)	0.0354 (12)	
C3	0.2204 (3)	0.4548 (5)	0.6687 (10)	0.0403 (12)	
C4	0.2487 (2)	0.5563 (5)	0.6445 (8)	0.0413 (14)	
C5	0.2937 (3)	0.5551 (5)	0.6073 (8)	0.0462 (16)	
C6	0.3146 (2)	0.4447 (6)	0.5866 (9)	0.0467 (15)	
H6	0.3451	0.4398	0.5603	0.056*	
C7	0.2887 (2)	0.3417 (5)	0.6061 (8)	0.0412 (14)	
C8	0.2665 (3)	0.1512 (6)	0.6131 (9)	0.0485 (17)	
C9	0.1720 (2)	0.4603 (6)	0.7129 (10)	0.0507 (17)	
H9A	0.1729	0.5172	0.8038	0.076*	
H9B	0.1635	0.3814	0.7483	0.076*	
H9C	0.1481	0.4858	0.6154	0.076*	
C10	0.3211 (3)	0.6695 (7)	0.5822 (11)	0.075 (3)	
H10A	0.3019	0.7171	0.4926	0.112*	
H10B	0.3514	0.6482	0.5526	0.112*	
H10C	0.3275	0.7158	0.6856	0.112*	
C11	0.2774 (3)	0.0181 (6)	0.5924 (12)	0.071 (3)	
H11A	0.2569	−0.0305	0.6468	0.107*	
H11B	0.3109	0.0022	0.6436	0.107*	
H11C	0.2715	−0.0017	0.4737	0.107*	
C12	0.1395 (2)	0.1660 (6)	0.4814 (9)	0.0512 (15)	
H12A	0.1524	0.1130	0.4072	0.077*	
H12B	0.1405	0.2483	0.4431	0.077*	
H12C	0.1065	0.1437	0.4806	0.077*	
C13	0.4940 (3)	0.3120 (8)	0.3690 (11)	0.064 (2)	
C14	0.4821 (3)	0.1834 (6)	0.3652 (8)	0.0507 (17)	
C15	0.5031 (3)	0.0711 (7)	0.3397 (11)	0.0552 (16)	
C16	0.4782 (3)	−0.0334 (7)	0.3580 (10)	0.0565 (19)	
C17	0.4301 (3)	−0.0306 (7)	0.4013 (10)	0.059 (2)	
C18	0.4098 (3)	0.0785 (7)	0.4214 (10)	0.060 (2)	
H18	0.3788	0.0834	0.4447	0.072*	
C19	0.4362 (2)	0.1824 (7)	0.4064 (9)	0.0511 (17)	
C20	0.4563 (3)	0.3746 (8)	0.4063 (11)	0.065 (2)	
C21	0.5530 (3)	0.0662 (10)	0.2927 (12)	0.084 (3)	
H21A	0.5767	0.0361	0.3877	0.126*	
H21B	0.5622	0.1462	0.2639	0.126*	
H21C	0.5515	0.0131	0.1971	0.126*	
C22	0.4033 (3)	−0.1465 (8)	0.4158 (11)	0.079 (3)	
H22A	0.3716	−0.1282	0.4355	0.118*	
H22B	0.4211	−0.1936	0.5089	0.118*	
H22C	0.4001	−0.1920	0.3123	0.118*	
C23	0.4473 (4)	0.5046 (8)	0.4241 (14)	0.085 (3)	
H23A	0.4274	0.5347	0.3204	0.128*	
H23B	0.4778	0.5473	0.4477	0.128*	
H23C	0.4309	0.5168	0.5158	0.128*	

### Atomic displacement parameters (Å^2^)

**Table:**

	*U*^11^	*U*^22^	*U*^33^	*U*^12^	*U*^13^	*U*^23^
Br1	0.0783 (5)	0.0482 (3)	0.0674 (4)	0.0096 (4)	0.0070 (3)	−0.0056 (3)
Br2	0.0915 (7)	0.0859 (5)	0.0720 (6)	0.0122 (5)	0.0022 (5)	−0.0039 (4)
S1	0.0518 (11)	0.0758 (13)	0.0445 (10)	−0.0326 (9)	0.0081 (8)	0.0092 (8)
S2A	0.026 (2)	0.063 (4)	0.063 (4)	−0.006 (2)	0.000 (2)	0.000 (3)
O4A	0.067 (8)	0.048 (6)	0.085 (10)	−0.015 (6)	0.011 (7)	0.037 (6)
C24A	0.044 (4)	0.080 (4)	0.056 (5)	−0.009 (3)	−0.001 (3)	−0.005 (3)
S2B	0.075 (3)	0.112 (5)	0.053 (3)	−0.062 (3)	0.006 (2)	0.018 (3)
O4B	0.065 (6)	0.163 (11)	0.075 (7)	−0.021 (6)	0.027 (5)	0.026 (7)
C24B	0.044 (4)	0.080 (4)	0.056 (5)	−0.009 (3)	−0.001 (3)	−0.005 (3)
O1	0.040 (3)	0.041 (2)	0.066 (3)	0.0064 (17)	0.013 (2)	0.0089 (19)
O2	0.091 (5)	0.082 (4)	0.103 (5)	−0.044 (4)	−0.015 (4)	0.058 (4)
O3	0.038 (3)	0.068 (3)	0.075 (4)	−0.005 (2)	0.005 (2)	0.004 (2)
C1	0.035 (4)	0.040 (3)	0.036 (3)	−0.013 (2)	0.009 (3)	0.006 (2)
C2	0.024 (3)	0.046 (3)	0.033 (3)	−0.008 (2)	0.000 (2)	0.002 (2)
C3	0.029 (3)	0.055 (3)	0.034 (3)	−0.006 (3)	0.000 (2)	−0.001 (3)
C4	0.041 (4)	0.045 (3)	0.036 (3)	−0.009 (3)	0.005 (3)	0.000 (2)
C5	0.049 (4)	0.044 (3)	0.043 (4)	−0.013 (3)	0.003 (3)	−0.002 (2)
C6	0.030 (3)	0.059 (4)	0.052 (4)	−0.014 (3)	0.010 (3)	0.004 (3)
C7	0.037 (3)	0.041 (3)	0.044 (3)	−0.005 (2)	0.006 (3)	0.004 (2)
C8	0.051 (4)	0.043 (4)	0.045 (4)	−0.006 (3)	−0.003 (3)	0.003 (3)
C9	0.041 (4)	0.058 (4)	0.055 (4)	0.000 (3)	0.015 (3)	−0.008 (3)
C10	0.069 (6)	0.061 (4)	0.090 (6)	−0.035 (4)	0.010 (5)	0.016 (4)
C11	0.078 (6)	0.045 (4)	0.082 (6)	−0.005 (4)	−0.002 (5)	0.007 (4)
C12	0.038 (4)	0.058 (3)	0.056 (4)	−0.012 (3)	0.008 (3)	−0.007 (3)
C13	0.043 (5)	0.084 (5)	0.058 (5)	−0.020 (3)	−0.004 (4)	0.016 (4)
C14	0.042 (4)	0.069 (4)	0.040 (4)	−0.021 (3)	0.004 (3)	0.009 (3)
C15	0.043 (4)	0.087 (5)	0.035 (3)	−0.005 (4)	0.008 (3)	0.008 (4)
C16	0.046 (4)	0.070 (4)	0.046 (4)	−0.001 (3)	−0.005 (3)	0.003 (3)
C17	0.047 (5)	0.066 (4)	0.061 (5)	−0.017 (3)	0.003 (4)	0.011 (3)
C18	0.038 (4)	0.072 (5)	0.067 (5)	−0.022 (3)	0.007 (3)	0.009 (3)
C19	0.029 (3)	0.070 (4)	0.051 (4)	−0.015 (3)	0.001 (3)	0.007 (3)
C20	0.047 (4)	0.081 (5)	0.066 (5)	−0.014 (4)	0.006 (4)	0.006 (4)
C21	0.055 (5)	0.132 (9)	0.069 (6)	−0.004 (5)	0.023 (4)	−0.006 (5)
C22	0.073 (6)	0.089 (6)	0.068 (5)	−0.039 (5)	0.004 (4)	0.014 (4)
C23	0.096 (7)	0.070 (6)	0.089 (7)	−0.019 (5)	0.018 (6)	−0.001 (5)

### Geometric parameters (Å, °)

**Table:**

Br1—C4	1.941 (6)	C9—H9C	0.9600
Br2—C16	1.871 (8)	C10—H10A	0.9600
S1—O2	1.499 (7)	C10—H10B	0.9600
S1—C1	1.783 (6)	C10—H10C	0.9600
S1—C12	1.788 (7)	C11—H11A	0.9600
S2A—O4A	1.102 (13)	C11—H11B	0.9600
S2A—C24A	1.761 (10)	C11—H11C	0.9600
S2A—C13	1.848 (10)	C12—H12A	0.9600
C24A—H24A	0.9600	C12—H12B	0.9600
C24A—H24B	0.9600	C12—H12C	0.9600
C24A—H24C	0.9600	C13—C20	1.352 (14)
S2B—O4B	1.044 (11)	C13—C14	1.454 (10)
S2B—C13	1.801 (10)	C14—C19	1.401 (10)
O1—C7	1.372 (7)	C14—C15	1.405 (11)
O1—C8	1.392 (9)	C15—C16	1.372 (10)
O3—C20	1.344 (10)	C15—C21	1.530 (12)
O3—C19	1.375 (10)	C16—C17	1.469 (12)
C1—C8	1.328 (10)	C17—C18	1.355 (11)
C1—C2	1.462 (7)	C17—C22	1.498 (10)
C2—C7	1.389 (9)	C18—C19	1.383 (9)
C2—C3	1.413 (9)	C18—H18	0.9300
C3—C4	1.411 (9)	C20—C23	1.466 (12)
C3—C9	1.479 (11)	C21—H21A	0.9600
C4—C5	1.362 (10)	C21—H21B	0.9600
C5—C6	1.375 (9)	C21—H21C	0.9600
C5—C10	1.513 (8)	C22—H22A	0.9600
C6—C7	1.375 (8)	C22—H22B	0.9600
C6—H6	0.9300	C22—H22C	0.9600
C8—C11	1.514 (10)	C23—H23A	0.9600
C9—H9A	0.9600	C23—H23B	0.9600
C9—H9B	0.9600	C23—H23C	0.9600
			
O2—S1—C1	108.4 (4)	S1—C12—H12A	109.5
O2—S1—C12	107.1 (4)	S1—C12—H12B	109.5
C1—S1—C12	96.4 (3)	H12A—C12—H12B	109.5
O4A—S2A—C24A	138.7 (9)	S1—C12—H12C	109.5
O4A—S2A—C13	120.3 (8)	H12A—C12—H12C	109.5
C24A—S2A—C13	97.6 (5)	H12B—C12—H12C	109.5
O4B—S2B—C13	130.4 (7)	C20—C13—C14	108.3 (7)
C7—O1—C8	105.1 (5)	C20—C13—S2B	132.0 (7)
C20—O3—C19	107.4 (7)	C14—C13—S2B	119.6 (8)
C8—C1—C2	107.2 (5)	C20—C13—S2A	112.6 (7)
C8—C1—S1	125.6 (4)	C14—C13—S2A	138.9 (8)
C2—C1—S1	127.2 (5)	C19—C14—C15	117.8 (6)
C7—C2—C3	120.3 (5)	C19—C14—C13	102.9 (7)
C7—C2—C1	104.1 (5)	C15—C14—C13	139.3 (7)
C3—C2—C1	135.7 (6)	C16—C15—C14	118.8 (7)
C4—C3—C2	112.8 (7)	C16—C15—C21	120.9 (8)
C4—C3—C9	125.2 (6)	C14—C15—C21	120.3 (7)
C2—C3—C9	122.0 (5)	C15—C16—C17	121.8 (7)
C5—C4—C3	127.0 (6)	C15—C16—Br2	120.9 (6)
C5—C4—Br1	117.8 (4)	C17—C16—Br2	117.3 (5)
C3—C4—Br1	115.2 (5)	C18—C17—C16	118.6 (6)
C4—C5—C6	118.4 (5)	C18—C17—C22	121.0 (8)
C4—C5—C10	123.1 (6)	C16—C17—C22	120.3 (8)
C6—C5—C10	118.5 (7)	C17—C18—C19	118.4 (7)
C7—C6—C5	117.7 (6)	C17—C18—H18	120.8
C7—C6—H6	121.1	C19—C18—H18	120.8
C5—C6—H6	121.1	O3—C19—C18	124.8 (7)
O1—C7—C6	124.5 (6)	O3—C19—C14	110.7 (6)
O1—C7—C2	111.7 (5)	C18—C19—C14	124.5 (8)
C6—C7—C2	123.7 (6)	O3—C20—C13	110.6 (7)
C1—C8—O1	112.0 (5)	O3—C20—C23	116.5 (8)
C1—C8—C11	136.1 (7)	C13—C20—C23	132.8 (8)
O1—C8—C11	111.9 (7)	C15—C21—H21A	109.5
C3—C9—H9A	109.5	C15—C21—H21B	109.5
C3—C9—H9B	109.5	H21A—C21—H21B	109.5
H9A—C9—H9B	109.5	C15—C21—H21C	109.5
C3—C9—H9C	109.5	H21A—C21—H21C	109.5
H9A—C9—H9C	109.5	H21B—C21—H21C	109.5
H9B—C9—H9C	109.5	C17—C22—H22A	109.5
C5—C10—H10A	109.5	C17—C22—H22B	109.5
C5—C10—H10B	109.5	H22A—C22—H22B	109.5
H10A—C10—H10B	109.5	C17—C22—H22C	109.5
C5—C10—H10C	109.5	H22A—C22—H22C	109.5
H10A—C10—H10C	109.5	H22B—C22—H22C	109.5
H10B—C10—H10C	109.5	C20—C23—H23A	109.5
C8—C11—H11A	109.5	C20—C23—H23B	109.5
C8—C11—H11B	109.5	H23A—C23—H23B	109.5
H11A—C11—H11B	109.5	C20—C23—H23C	109.5
C8—C11—H11C	109.5	H23A—C23—H23C	109.5
H11A—C11—H11C	109.5	H23B—C23—H23C	109.5
H11B—C11—H11C	109.5		
			
O2—S1—C1—C8	14.9 (8)	O4A—S2A—C13—C14	139.6 (14)
C12—S1—C1—C8	−95.6 (7)	C24A—S2A—C13—C14	−57.7 (11)
O2—S1—C1—C2	−165.0 (6)	O4A—S2A—C13—S2B	115.7 (17)
C12—S1—C1—C2	84.5 (6)	C24A—S2A—C13—S2B	−81.5 (10)
C8—C1—C2—C7	−0.7 (7)	C20—C13—C14—C19	−1.2 (9)
S1—C1—C2—C7	179.2 (5)	S2B—C13—C14—C19	−177.5 (6)
C8—C1—C2—C3	179.8 (8)	S2A—C13—C14—C19	173.1 (9)
S1—C1—C2—C3	−0.3 (11)	C20—C13—C14—C15	−178.4 (9)
C7—C2—C3—C4	2.2 (9)	S2B—C13—C14—C15	5.3 (14)
C1—C2—C3—C4	−178.3 (7)	S2A—C13—C14—C15	−4.2 (18)
C7—C2—C3—C9	−178.6 (7)	C19—C14—C15—C16	−0.7 (11)
C1—C2—C3—C9	0.9 (12)	C13—C14—C15—C16	176.2 (9)
C2—C3—C4—C5	−2.3 (11)	C19—C14—C15—C21	179.4 (7)
C9—C3—C4—C5	178.5 (7)	C13—C14—C15—C21	−3.6 (15)
C2—C3—C4—Br1	177.7 (5)	C14—C15—C16—C17	0.7 (11)
C9—C3—C4—Br1	−1.5 (10)	C21—C15—C16—C17	−179.4 (8)
C3—C4—C5—C6	1.5 (11)	C14—C15—C16—Br2	−176.9 (6)
Br1—C4—C5—C6	−178.6 (4)	C21—C15—C16—Br2	3.0 (10)
C3—C4—C5—C10	179.5 (7)	C15—C16—C17—C18	0.9 (11)
Br1—C4—C5—C10	−0.5 (9)	Br2—C16—C17—C18	178.6 (5)
C4—C5—C6—C7	−0.5 (9)	C15—C16—C17—C22	178.4 (7)
C10—C5—C6—C7	−178.6 (6)	Br2—C16—C17—C22	−3.9 (10)
C8—O1—C7—C6	−178.8 (6)	C16—C17—C18—C19	−2.4 (11)
C8—O1—C7—C2	−1.2 (7)	C22—C17—C18—C19	−179.9 (7)
C5—C6—C7—O1	177.9 (6)	C20—O3—C19—C18	178.4 (7)
C5—C6—C7—C2	0.6 (10)	C20—O3—C19—C14	−3.1 (8)
C3—C2—C7—O1	−179.2 (6)	C17—C18—C19—O3	−179.2 (7)
C1—C2—C7—O1	1.2 (7)	C17—C18—C19—C14	2.6 (11)
C3—C2—C7—C6	−1.5 (10)	C15—C14—C19—O3	−179.4 (6)
C1—C2—C7—C6	178.8 (6)	C13—C14—C19—O3	2.6 (8)
C2—C1—C8—O1	0.0 (8)	C15—C14—C19—C18	−1.0 (11)
S1—C1—C8—O1	−179.9 (5)	C13—C14—C19—C18	−178.9 (7)
C2—C1—C8—C11	−178.3 (8)	C19—O3—C20—C13	2.2 (9)
S1—C1—C8—C11	1.8 (13)	C19—O3—C20—C23	−179.3 (7)
C7—O1—C8—C1	0.7 (8)	C14—C13—C20—O3	−0.6 (10)
C7—O1—C8—C11	179.5 (6)	S2B—C13—C20—O3	175.1 (7)
O4B—S2B—C13—C20	−94.9 (16)	S2A—C13—C20—O3	−176.5 (6)
O4B—S2B—C13—C14	80.3 (15)	C14—C13—C20—C23	−178.7 (9)
O4B—S2B—C13—S2A	−117.4 (19)	S2B—C13—C20—C23	−3.1 (16)
O4A—S2A—C13—C20	−46.3 (13)	S2A—C13—C20—C23	5.3 (13)
C24A—S2A—C13—C20	116.4 (7)		

### Hydrogen-bond geometry (Å, °)

**Table:**

*D*—H···*A*	*D*—H	H···*A*	*D*···*A*	*D*—H···*A*
C12—H12A···O2^i^	0.96	2.34	3.298 (9)	179
C12—H12C···O4B^ii^	0.96	2.42	3.168 (12)	134
C24A—H24A···O4A^iii^	0.96	2.35	3.199 (13)	148
C9—H9A···Cg2^iii^	0.96	3.30	3.943 (9)	124

Symmetry codes: (i) *x*, −*y*, *z*−1/2; (ii) *x*−1/2, −*y*+1/2, *z*+1/2; (iii) *x*, −*y*+1, *z*+1/2.
